# Annotations

**Published:** 1904-02-13

**Authors:** 


### ANNOTATIONS.

### Dust Collecting in Germany.

It seems that the cholera epidemic of 1892 was a blessing in disguise to Germany, for it set people to devising better methods of collecting dust and other refuse which might convey the germs of infection, and in consequence this is a part of municipal work which is now better done in Germany than in England. The municipal regulations of Berlin insist that refuse, ashes, and all that is generally though inaccurately summarised by the term "dust" should be carried through the streets in air-tight receptacles. This has set ingenuity to work in the inventing of both dustbins and dust-carts, and by means of suitable arrangements in the latter, it has been found possible to take away household refuse without defiling the streets with either the offensive smells or the infectious dust from which we suffer here. More perfect than any dust-cart is the "substitution system." By this plan each householder has two dustbins, of which, however, only one is in his actual possession at a time. This, when full, is carried away bodily in the dust-cart, in which has been brought back the empty one for use until the cart calls again. The disadvantage of this otherwise admirable system is that the extra weight and bulk of the dustbins involves the employment of a larger number of carts than would otherwise be necessary. To avoid this trouble, bags have been used instead of bins. These bags are made of asbestos, to avoid any risk of burning from hot cinders. Unfortunately they are not only expensive, but soon wear out. However, if motor instead of horse traction were used the objection both to the substitution system and to the employment of heavy but air-tight dust-carts might be to some extent obviated. In any case, the attempt of the German authorities to improve methods of dust removal is a thing which our municipalities might do well both to study and to imitate.

### Standardised Preparations.

The virtue of accuracy may be said to make a special appeal in connection with medicinal substances and preparations. When questions of health and life are at stake it is obvious that the means taken to secure these should if possible be in fact what they profess to be in name. The National Pharmacopœia makes some contribution to this end by fixing standard definitions for the terms in common use in physicians' prescriptions. But it leaves many loopholes and weak places, some of which in the present state of our knowledge can hardly be repaired. There are others, however, which are not in this position, and it is well that to these attention should be given. As we write we have before us the report of the analyst to a large and well-known firm of manufacturing chemists and from its pages we find that an extract of belladonna made last year was found to contain nearly 50 per cent. less alkaloid than the extract prepared in the previous year. Indeed it is obvious that preparations of this order, galenicals as they are called, though made strictly in accordance with the official instructions, may vary considerably in the proportion of the active principle they contain. Such a state of matters carries risk both to the physician and the patient and it cannot be doubted that this variation and uncertainty are largely responsible for the varying and sometimes contradictory results which attend the actions of drugs. The remedy is of course to insist upon the introduction into the "Pharmacopœia" of quantitative tests by which a definite standard of value may be determined. Preparations in other words must be standardised. This is now possible in many cases thanks largely to the enterprise of certain firms of manufacturing chemists, and the practice ought to receive practical recognition. By securing rigid accuracy in the percentage of active principles the physician is enabled to use his remedies with more confidence and precision, and both he and his patient reap an advantage.

### The Guild of "The Poor Things."

This is the name of a Bristol charity. The quaintly pathetic title implies that the members of the guild are lame, or blind, or in some such way afflicted that the world will be inclined to pity them as "poor things." The intention is to lift them out of the humiliation suggested by such pity. Some of the members are rich and some are poor, but all are expected to fit themselves to subscribe to the motto of the guild, "Laetus sorte mea"—"happy in my lot"—an inscription taken from Mrs. Ewing's "Story of a Short Life." It cannot be said that all attain to this summit of contentment, but as much as may be they are helped by friends who teach them various kinds of work, both recreative and profitable, arrange country holidays for them, and gather them together for study and amusement during the winter months. At these gatherings, says the report, "There are scarlet badges and gay flags; there are bright faces; there are brave hearts; there are merry little children unconscious of the sadness of their lot." The adults cannot be so gay as the children, but even they find their burden lightened by the efforts of the guild. One young man of four-and-twenty, already quite deaf and getting gradually blind, declared when he first came in contact with the Guild that death was all he desired; but when a place had been found where he could learn chair-caning and he was able to leave his lonely attic and live with friends who would sympathise with and care for him, he wrote, in his scarcely legible writing, "I have been much more happier since I have been a member of the Guild." "Much more happier" also is the half-paralysed boy, once a burden to his family, who has been set up with a donkey and cart to sell coal, and is a bread-winner in his degree, and the girl with spinal disease who has learned to make straw dinner-mats while lying on her back, and the man who, having learned to make chairs, has been able to do without parish help for more than a year. Several of the Guild's clients have thus been able either to do without or to give up parish doles. Of course, all cannot be thus lifted above the need of material help, and the Guild must be content with making their lot brighter than it was; and all the advice that the helpers can give is contained in the words of Mazzini, which are placed at the head of the report: "When you cannot have Victory, salute and bless Martyrdom." But that serves to lead up to the succeeding sentence, "the angels of Martyrdom and Victory are brothers."

